# Influence of Block of NF-Kappa B Signaling Pathway on Oxidative Stress in the Liver Homogenates

**DOI:** 10.1155/2013/308358

**Published:** 2013-03-14

**Authors:** Paulina Kleniewska, Aleksandra Piechota-Polanczyk, Lukasz Michalski, Marta Michalska, Ewa Balcerczak, Marta Zebrowska, Anna Goraca

**Affiliations:** ^1^Experimental and Clinical Physiology, Department of Cardiovascular Physiology, Medical University of Lodz, 6/8 Mazowiecka Street, 92-215 Lodz, Poland; ^2^Department of Biochemical Pharmacy, Department of Pharmacy, Medical University of Lodz, 1 Muszynskiego Street, 91-151 Lodz, Poland; ^3^Laboratory of Molecular Diagnostic and Pharmacogenomics, Department of Biochemical Pharmacy, Medical University of Lodz, 1 Muszynskiego Street, 91-151 Lodz, Poland

## Abstract

The aim of the present study was to assess whether BAY 11-7082, a nuclear factor-kappaB (NF-**κ**B) inhibitor, influences the level of reactive oxygen species (ROS), tumor necrosis factor alpha (TNF-**α**), and NF-**κ**B related signaling pathways in the liver. The animals were divided into 4 groups: I: saline; II: saline + endothelin-1 (ET-1) (1.25 **μ**g/kg b.w., i.v.); III: saline + ET-1 (12.5 **μ**g/kg b.w., i.v.); and IV: BAY 11-7082 (10 mg/kg b.w., i.v.) + ET-1 (12.5 **μ**g/kg b.w., i.v.). Injection of ET-1 alone at a dose of 12.5 **μ**g/kg b.w. showed a significant (*P* < 0.001) increase in thiobarbituric acid reactive substances (TBARS) and hydrogen peroxide (H_2_O_2_) level and decrease (*P* < 0.01) in GSH level (vs. control). ET-1 administration slightly downregulated gene expression of p65 of NF-**κ**B but potently and in a dose-dependent way downregulated p21-cip gene expression in the liver. BAY 11-7082 significantly decreased TBARS (*P* < 0.001), H_2_O_2_ (*P* < 0.01) and improved the redox status (*P* < 0.05), compared to ET-1 group. The concentration of TNF-**α** was increased in the presence of ET-1 (*P* < 0.05), while BAY 11-7082 decreased TNF-**α** concentration (*P* < 0.01). Inhibition of IkB**α** before ET-1 administration downregulated gene expression of p21-cip but had no effect on p65.

## 1. Introduction

Endothelin-1 (ET-1) is a potent vasoconstrictor polypeptide containing 21 amino acids. It was originally isolated from the culture supernatant of porcine aortic endothelial cells by Yanagisawa et al. [1]. However, ET-1 has been shown to exert a variety of biological actions in many different cell types including endothelial cells [2, 3]. The known hepatic cellular sources of ET-1 is the liver sinusoidal endothelial cells, the hepatic stellate cells, and the Kupffer cells [4].

ET-1 triggers its biological action after binding to ET_A_, ET_B1_, or ET_B2_ receptor. Each of those receptors is found in the liver with predominance of ET_B_ over ET_A_ receptor [5–9]. ET-1 acting *via* the ET_A_ and ET_B2_ receptor causes a contraction of effector cells. On the contrary, ET-1 binding to ET_B1_ receptor on endothelial cells causes NO^−^ dependent relaxation of adjacent vascular smooth muscle cells and peritocytes.

Studies indicated that blood and tissue levels of ET-1 were enhanced in humans during liver inflammation [10–14] and in animal models of liver ischemia-reperfusion [15, 16]. During pathological conditions, ET-1 production shifts from the sinusoidal endothelial cells to the hepatic stellate cells [16, 17]. Some authors speculate that the hepatic stellate cells are the primary “target” of ET-1 in the liver [8, 18]. Moreover, stellate cells, particularly during the liver injury, externalized far more ET-1 receptors than other liver cells [8, 18]. It has been indicated that ET-1 causes a contraction of the isolated stellate cells in culture [4, 19]. Moreover, systemic or intraportal infusion of ET-1 narrows the sinusoidal lumen in the isolated perfused liver [20] as well as in the liver with afferent nerves. This action is speculated to be mediated *via* phospholipase C activation [21–23].

Apart from its vasomotor action, ET-1-induced signaling *via* ET_A_ receptor is characterized by a rapid induction of nuclear factor-kappa B (NF-*κ*B) p65 subunit/mitogen activated phosphokinase p38 transcription complex. This molecular signaling leads to releasing of reactive oxygen species (ROS) and proinflammatory cytokines like tumor necrosis factor alpha (TNF-*α*). TNF-*α* is known to cause increased production of ROS *via* NF-*κ*B [24] and NF-*κ*B dependent cell-cycle regulating pathways like p21-cip (cyclin dependant inhibitor) [25]. Expression of the p21-cip, a member of the Cip/Kip family, increases when cells are damaged. In addition p21 participates in DNA repair and apoptotic processes [26]. Furthermore, ROS accumulation under high TNF-*α* concentration increases the DNA-binding activity of NF-*κ*B required for ET-1 in endothelial cells [27]. Therefore, the release of proinflammatory cytokines and activation of NF-*κ*B and p21-cip may strongly intensify the inflammatory process and cell damage and contribute to the microvascular dysfunction and liver injury [14]. Moreover, TNF-*α*-induced ROS generation increases ET-1 gene transcription and ET-1 production [27, 28].

The aim of this study was to investigate whether pharmacological inhibition of NF-*κ*B activation, a major regulator of inflammatory response, by BAY 11-7082 ameliorates liver dysfunction induced by exogenous ET-1 infusion. 

## 2. Materials and Methods

### 2.1. Animals

The experiments were performed under urethane anesthesia on male Wistar rats weighing 260–280 g and aged 2-3 months. The rats were acquired from the Medical University of Lodz Animal Quarters and were housed in individual cages under standard laboratory conditions: 12/12 h light-dark cycle (light on at 7.00 a.m.) at 20 ± 2°C ambient temperature and 55 ± 5% air humidity. All animals received a standard laboratory diet and water *ad libitum*. All animals were maintained for 1 week in the laboratory for adaptation. The experimental procedures followed the guidelines for the care and use of laboratory animals, and were approved by the Medical University of Lodz Ethics Committee number 20/L418/2008. 

### 2.2. Experimental Design

Animals were randomly divided into four groups: (1) control group (*n* = 8): rats received i.v. two doses of 0.2 mL saline, 30 min apart; (2) and (3) endothelin-1 groups (*n* = 8): ET-1 at a dose of 1.25 *μ*g/kg b.w. and 12.5 *μ*g/kg b.w., respectively, was injected i.v. 60 min after saline administration; (4) BAY group (*n* = 8): BAY 11-7082 10 mg/kg b.w. was injected 60 min before ET-1 (12.5 *μ*g/kg b.w.) injection. 

### 2.3. Animal Preparations

Animals were anaesthetized by an intraperitoneal injection of 10% urethane (2 mL/100 g b.w.). When a sufficient level of anesthesia was achieved, the skin and subcutaneous tissues on the neck were cut and then a 2 cm-long polyethylene tube (2.00 mm O.D.) was inserted into the trachea. The right femoral vein was catheterized for drug infusion. 

### 2.4. Tissue Preparation and Collection of Samples

The animals were euthanized under anesthesia 5 hours after saline or ET-1 injection. The thoracic cavity was opened to remove the liver. Then the liver was cleaned of extraneous tissue. It was rinsed with cold isotonic saline, dried by blotting between two pieces of filter paper, and weighed on an electronic scale to estimate liver edema. The harvested liver was stored at −76°C for measurement of oxidative parameters.

### 2.5. Preparation of Homogenates

An accurately weighed portion of liver (50 mg) was homogenized in either 0.15 M KCl for the estimation of lipid peroxidation and concentration of H_2_O_2_ or in 5% SSA for the estimation of glutathione. Homogenates were centrifuged at 10,000 × g, 10 min, and 4°C for glutathione measurement or at 3,500 rpm, +4°C, 15 min for lipid peroxidation assay. The resulting supernatant was used for biochemical analyses immediately.

### 2.6. Determination of Lipid Peroxidation

The formation of thiobarbituric acid-reactive substances (TBARS) was used to quantify the degree of lipid peroxidation in tissues. The peroxidation product content in liver homogenates was assayed as TBARS and TBA-reactive substance concentration in the butanol layer were measured spectrofluorometrically using an LS-50 Perkin Elmer Luminescence Spectrometer (Norwalk, CT, U.S.A.). Excitation was set at 515 nm and emission was measured at 546 nm. The TBARS concentration in the sample was calculated by the use of the regression equation: *Y* = 0.43(*X* − *Xo*) − 2.43, where *Y*= TBARS concentration (*μ*M); *X*, *Xo* = fluorescence intensity of the samples and control, respectively (arbitrary units: AU). The regression equation was prepared from triplicate assays of six increasing concentrations of tetramethoxypropane (range 0.01 to 50 *μ*M) as a standard for TBARS. Finally, the results were calculated for 50 mg of the liver tissue.

### 2.7. Determination of H_2_O_2_

The H_2_O_2_ concentration in homogenates was measured using HRP/HVA systems. Samples were incubated for 60 min at 37°C after which the enzymatic reaction was stopped by adding 0.1 M glycine-NaOH buffer (pH 12.0) with 25 mM EDTA. Excitation was set at 312 nm and emission was measured at 420 nm (Perkin Elmer Luminescence Spectrometer, Beaconsfield UK). The readings were converted into a value for H_2_O_2_ concentration using the regression equation: *Y* = 0.03615*X* − 0.081, where *Y* = H_2_O_2_ concentration in a homogenate (*μ*M); *X* = intensity of light emission at 420 nm for HRP + HVA assay reduced by HRP assay emission (arbitrary units: AU). The regression equation was prepared from three series of calibration experiments with 10 increasing H_2_O_2_ concentrations (range 10–1,000 *μ*M). The lowest H_2_O_2_ detection was 0.1 nM, with intra-assay variability not exceeding 2%.

### 2.8. Determination of GSH Levels

Total glutathione (tGSH), reduced glutathione (GSH), and oxidized glutathione (GSSG) were measured in the hepatic tissue homogenates. The total GSH content of the supernatant was measured in a 1 mL cuvette containing 0.7 mL of 0.2 mM NADPH, 0.1 mL of 0.6 mM DTNB, 0.150 mL of H_2_O, and 50 *μ*L of the sample. The cuvette with the mixture was incubated for 5 min at 37°C and then supplemented with 0.6 U of GR. The reaction kinetics was traced spectrophotometrically at 412 nm for 5 min by monitoring the increase in absorbance. GSSG concentration was determined in supernatant aliquots using the same protocol after optimization of pH to 6-7 with 1 M TEA and derivatization of endogenous GSH with 2-vinylpyridine (v : v). The reduced GSH level in the supernatant was calculated as the difference between total GSH and GSSG. The increments in absorbance at 412 nm were converted to GSH and GSSG concentrations using a standard curve (3.2–500 *μ*M of GSH for total GSH and 0.975–60 *μ*M of GSSG for GSSG). The results were expressed in *μ*M.

### 2.9. GSH/GSSG Redox Ratio

The redox ratio of the ET-1 or BAY treated/untreated samples was calculated by dividing the reduced glutathione content over the oxidized glutathione content of their respective samples.

### 2.10. Tumor Necrosis Factor-*α* Assay

The concentration of TNF-*α* in the liver homogenates was measured using enzyme-linked immunosorbent assay (ELISA) commercial Kit (Quantikine, R&D System, USA) according to the manufacturer's instructions. The results were read using a TEK Instruments EL340 BIO-spectrophotometer (Winooski VT, USA) (*λ* = 45 nm). The lowest limit of detection for TNF-*α* was 0.037 pg/mL. The proper TNF-*α* concentration was read from a standard curve and expressed in pg/mL. 

### 2.11. RNA Isolation and RT-PCR for p65 and p21

RNA was isolated by Total RNA Prep Plus Minicolumn Kit (A&A Biotechnology, Poland) based on RNA isolation method developed earlier [29]. For real-time PCR normalization UV absorbance was used to determine the amount of RNA added to a cDNA reaction. PCRs are then set up using cDNA derived from the same amount of input RNA. The isolated RNA has an A260/280 ratio of 1.6–1.8. Before the quantitative analysis of gene expression by real-time PCR reaction, the parameters were checked using qualitative PCR. PCR reaction mixture for PCR amplification consisted of a cDNA template, 0.5 *μ*M of each primer, 10 × AccuTaq Buffer, 0.5 U of AccuTaq LA DNA Polymerase Mix, 0.2 mM each dNTP, water to a final volume of 20 *μ*L. Negative control was included in each experiment (sample without a cDNA template).

Real-time PCR reactions were done using Rotorgene 6,000 (Corbett Life Science). The p65 gene and a reference Bactin were amplified parallel for each sample in separate wells, during the same PCR, the same experiment for pair of genes p21 and Bactin. The primers sequences for investigated genes p65 and p21 and also for reference gene Bactin were planned by using software Primer3: WWW primer tool (http://biotools.umassmed.edu/bioapps/primer3_www.cgi). The primers were as follows: Bactin (forward primer: 5′-gTg ggg CgC CCC Agg CAC CA-3′, reverse primer: 5′-CTC CTT AAT gTC ACg CAC gAT TTC-3′), p21-cip (forward primer 5′-TTg CAC TCT ggT gTC TgA gC-3′, reverse primer: 5′-AAT CTG TCA GGC TGG TCT GC-3′), p65 (forward primer. 5′-ACAACCCCTTCCAAGTTCCCT-3′, and reverse primer: 5′-TGGTCCCGTGAAATACACCT-3′).

Bactin was utilized as an internal positive control and as a normalizer for expression data correction. The thermal cycling conditions comprised an initial denaturation step at 95°C for 2 min, 35 cycles at 94°C for 30 s, 58°C (p65), 57°C (p21) for 30 s and 72°C for 30 s, and a final extension step at 72°C for 3 min. The relative levels of p21 and p65 expression were calculated as described previously [30]. 

### 2.12. Statistical Analysis

The data are presented as mean ± S.E.M. if not stated otherwise, from 8 animals in each group. The statistical analysis was performed by ANOVA followed by the Duncan's multiple range test as post hoc. *P* values lower than 0.05 were considered significant.

## 3. Results

### 3.1. Effect of ET-1 and BAY 11-7082 on Stress Oxidative Parameters and TNF-*α* Concentration

Changes in oxidative damage parameters are presented in Table 1. The administration of ET-1 at a dose of 12.5 *μ*g/kg b.w. resulted in a significant increase in TBARS (*P* < 0.001) and H_2_O_2_ (*P* < 0.001) concentrations as compared with the control group. 

BAY 11-7082 administered 1 hour before ET-1 (12.5 *μ*g/kg b.w., i.v.) significantly decreased TBARS (*P* < 0.001) and H_2_O_2_ (*P* < 0.01) levels as well as improved the redox status (*P* < 0.05) in the liver, compared to ET-1 (12.5 *μ*g/kg b.w.) group (Table 1). 

**Figure 1:**
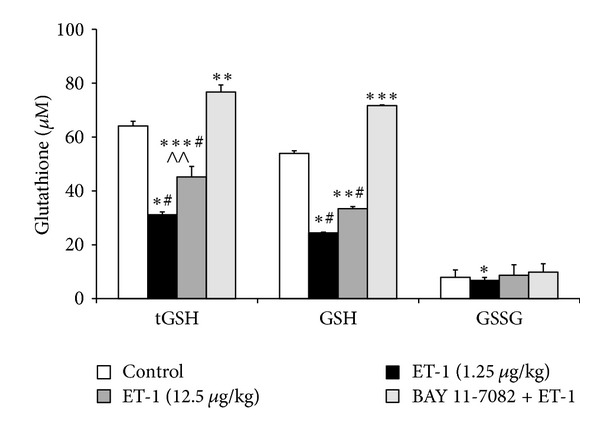
Liver status of the glutathione metabolism in the control group, the endothelin-1 groups, (1.25 and 12.5 *μ*g/kg/b.w.) and BAY 11-7082 (10 mg/kg b.w.) administered 1 hour before ET-1 (12.5 *μ*g/kg b.w.) group (*n* = 8, per group). Data is shown as mean ± S.E.M. ET-1-endothelin-1 at doses of 1.25 and 12.5 *μ*g/kg b.w.; BAY+ET-1-BAY 11-7082 (10 mg/kg b.w.) administered 1 hour before endothelin-1 (12.5 *μ*g/kg b.w.) group; tGSH-total glutathione; GSH-reduced glutathione; GSSG-oxidized glutathione.*P < 0.001,**P < 0.01, ***P < 0.02, and ****P < 0.05  *versus* saline; ^#^ *P* < 0.001, ^##^ *P* < 0.01, ^###^ *P* < 0.02, and ^####^ *P* < 0.05  *versus* BAY 11-7082 + ET-1; ^∧^ *P* < 0.001, ^∧∧^ *P* < 0.01  *versus* ET-1 (1.25 *μ*g/kg b.w).

Intravenous administration of endothelin-1 at both doses 1.25 or 12.5 *μ*g/kg b.w. resulted in a significant decrease in total glutathione (tGSH) (*P* < 0.001;  *P* < 0.02, resp.) and reduced glutathione (GSH) concentrations (*P* < 0.001; *P* < 0.01 resp.) as compared to saline-treated controls. Treatment of rats with BAY 11-7082 (10 mg/kg) and endothelin-1 (12.5 *μ*g/kg) significantly enhanced the endothelin-1-induced decrease in tGSH and GSH concentrations in comparison with endothelin-1 group in both doses: 1.25 and 12.5 *μ*g/kg b.w. (*P* < 0.001) (Figure 1).

The redox status (GSH/GSSG ratio), an oxidative stress indicator, was found to be significantly reduced in ET-1 (12.5 *μ*g/kg b.w.) group as compared to saline treated controls (*P* < 0.001) and significantly increased in BAY11-7082 + ET-1 groups as compared to ET-1 (12.5 *μ*g/kg b.w.) group (*P* < 0.05) (Table 1).

**Figure 2:**
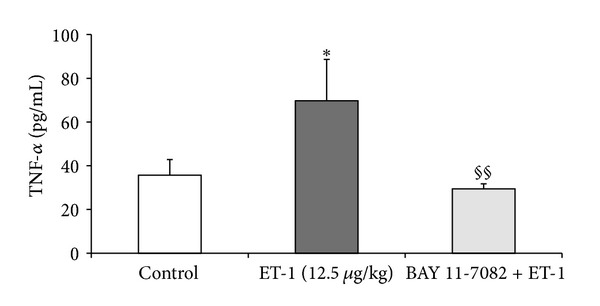
TNF-*α* concentration in liver homogenates in the control group, after endothelin-1 (1.25 and 12.5 *μ*g/kg b.w.) and BAY 11-7082 + endothelin-1 (10 mg/kg b.w. and 12.5 *μ*g/kg b.w., resp.) group. (*n* = 8, per group). Data is shown as mean ± S.E.M. ET-1-endothelin-1 at doses of 1.25 and 12.5 *μ*g/kg b.w.; BAY+ET-1-BAY 11-7082 (10 mg/kg b.w.) administered 1 hour before ET-1 (12.5 *μ*g/kg b.w.) group; TNF-*α*-tumor necrosis factor; ^§§^ *P* < 0.01  *versus* ET-1 (12.5 *μ*g/kg).

Figure 2 shows that 5 hours after ET-1 administration the liver concentration of TNF-*α* was significantly increased *versus* control group (*P* < 0.05). By contrast, the level of hepatic TNF-alpha was significantly diminished in the BAY11-7082 + ET-1 group as compared to ET-1 group (29.48 ± 2.24 pg/mL *versus *69.75 ± 18.89 pg/mL; *P* < 0.01).

### 3.2. Effect of ET-1 and BAY 11-7082 on p21 and p65 Gene Expression

**Figure 3:**
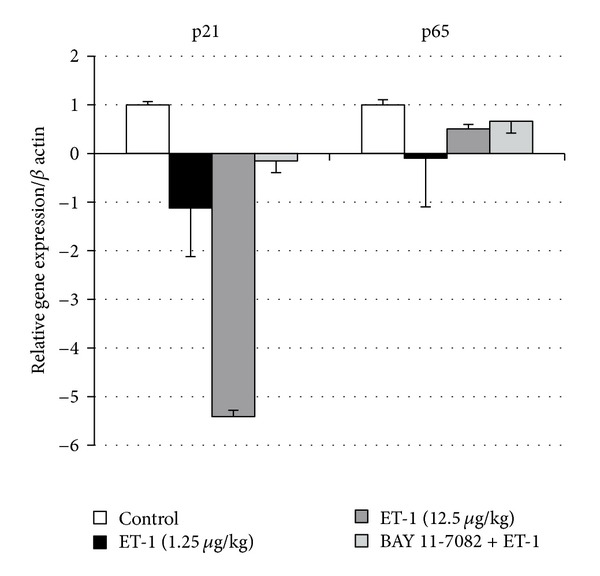
The p65 and p21-cip relative gene expression in the control group, after endothelin-1 (1.25 and 12.5 *μ*g/kg b.w.) and BAY 11-7082 + endothelin-1 (10 mg/kg b.w. and 12.5 *μ*g/kg b.w., resp.) group. (*n* = 4, per group). Data is shown as mean ± S.E.M.

All investigated cases for both genes were checked by qualitative PCR. In all cases expression of p21 and p65 was observed. Our results indicated a downregulation of p21-cip gene expression after ET-1 administration in the liver. The effect of ET-1 was dose-dependent and the 10-fold higher ET-1 dose caused a 5-fold decrease in p21-cip gene expression when compared to the control. The inhibition of NF-*κ*B with BAY 11-7082 did not entirely prevent ET-1-induced downregulation of p21-cip; however, its expression was markedly diminished (Figure 3).

Analysis of p65 mRNA level is presented in Figure 3. The ET-1 infusion had a biphasic effect on p65 mRNA level. The lower dose of ET-1 (1.25 *μ*g/kg) strongly downregulated p65 mRNA level, while 10-fold greater ET-1 dose exerted a weaker downregulated effect. Inhibition of I*κ*-B*α* degradation with BAY 11-7082 before ET-1 administration prevented changes in p65 mRNA level, therefore, indicating a successful inhibition of NF-*κ*B signaling pathway.

## 4. Discussion

In this study, the administration of ET-1 to rats resulted in a development of oxidative damage in the liver tissue accompanied by a change in the gene expression of p65 subunit of NF-*κ*B and a dose-dependent down-regulation of p21-cip. Oxidative stress was reflected by an increase in the concentrations of TBARS, H_2_O_2_, and TNF-*α*, as well as a decrease in the redox status.

The increase in TBARS concentration in our study was a result of increased production of ROS which in turn led to an excessive peroxidation of polyunsaturated fatty acids, causing liver damage [9]. It has been previously reported that ET-1 stimulates ROS production by activation of neutrophils and Kupffer cells [9, 21]. ROS cause a dose-dependent formation of large intracellular gaps and reduce the diameter of the remaining endothelial fenestration. These events lead to hypoperfusion by deterioration of microcirculation, therefore contributing to hepatotoxic injury [14, 31, 32]. Moreover, microcirculatory disturbances are a reason for insufficient energy supply, alteration in the mitochondrial redox state and subsequent decline in hepatic tissue oxygenation [33]. In addition, ROS-induced liver injury is mediated by the direct effects of ROS on signal transduction pathway that is activated through oxidation kinases and phosphatases [34]. Here, the mitogen-activated protein kinases (MAPK) and NF-*κ*B pathways play a particular role. Our results support the hypothesis that the lipid peroxidation process causes liver cell damage after ET-1 infusion.

The increasing formation of ROS in our study was indicated by an increased H_2_O_2_ concentration in the liver homogenates. H_2_O_2_, which is a major component of intracellular ROS during many physiological and pathological processes, causes oxidative damage of tissues. H_2_O_2_ is formed during the superoxide anion dismutation. This radical possesses a low molecular weight; therefore, it easily penetrates the lipid membrane causing lipid peroxidation and disturbance in lipid homeostasis. The basic enzymes that regulate the intracellular H_2_O_2_ concentration are catalase and glutathione peroxidase [35]. During oxidative stress, the concentration of those enzymes decline resulting in an intense H_2_O_2_ conversion to toxic hydroxyl radicals. This effect supports our results for an increase in H_2_O_2_ production after ET-1 administration. It has been previously shown that rats treated with H_2_O_2_ had a higher liver lipid peroxidation level and increased protein damage [36–38]. 

The most important intracellular non-protein thiol compound is reduced glutathione (GSH), which plays a major role in the protection of cells and tissue structures from oxidative injury [39]. In our study the decrease in GSH concentration after ET-1 administration indicated the development of oxidative stress in the liver cells.

The results of our studies reveal that ET-induced oxidative stress resulted in an enhanced TNF-*α* content in the liver homogenates. Kim et al. [40] showed that TNF-*α* elevation can be a marker of hepatitis B or C as its concentration increases greatly during liver injuries. Moreover, TNF-*α* stimulates the production of other cytokines that acting together leads to death of hepatocytes. ET-1-induced oxidative stress and TNF-*α* excretion activate NF-*κ*B, which in turn increases the release of TNF-*α*, thereby contributing to liver injury [41–43]. 

In our study ET-1-induced oxidative stress and ameliorated TNF-*α* level were accompanied by changes in the gene expression of p65 of NF-*κ*B in the liver. We observed a downregulation of p65 gene expression five hours after ET-1 administration at a dose of 1.25 *μ*g/kg and almost no inhibiting effect of the 10-fold higher ET-1 dose on p65 gene. Those results may be due to the fact of an early downregulation of NF-*κ*B signaling pathway, as we showed a strong concomitant inhibition of NF-*κ*B-regulated p21-cip gene [44]. Moreover, it was indicated previously that both ET-1 and TNF-*α* lead to a rapid and prolonged stimulation of p65 of NF-*κ*B subunit and stronger binding to native preproET-1 promoter [45]. Also Gallois et al. [46] presented that ET-1 increases the formation of p50 and p65 of NF-*κ*B DNA binding complexes and it increases the degradation of IkB*α* in hepatic stellate cells. Therefore, those changes may result in ameliorated ET-1 production and stronger expression of numerous cytokines, such as TNF-*α* [47].

In our study, we observed a dose-dependent effect of ET-1 regarding NF-*κ*B-regulated p21-cip gene. When administrated at a dose of 1.25 *μ*g/kg ET-1 downregulated p21 and this inhibition was stronger for a 10-times greater dose of ET-1. We speculate that this effect can be connected with ameliorated ROS and TNF-*α* concentration which had a negative feedback on the key cell cycle regulator and proapoptotic p21-cip pathway. Therefore, stronger oxidative stress observed after infusion of higher dose of ET-1 could have triggered a faster deprivation of gene expression for p21. Furthermore, an inhibition of p21 in hepatocytes was shown to be beneficial in mice model of cirrhosis leading to greater proliferation of hepatocytes which led to a larger liver mass and less architectural distortion [25]. However, further evaluation of p21-cip signaling in liver is needed to precise the role of p21 in liver diseases.

In our study, pharmacological inhibition of NF-*κ*B signaling pathway, by IKK*β* inhibitor BAY 11-7082, significantly lowered TBARS and H_2_O_2_ concentration. The decrease in TBARS and H_2_O_2_ concentration was a result of the diminished ROS production in the liver tissue. It is in line with Calabrò et al. [48] who indicated that BAY 11-7082 reduced reactive oxygen species in human coronary artery endothelial cells. Cho et el. [49] indicated that BAY 11-7082 inhibited ROS production and increased GSH levels in Kupffer cells. While Zanotto-Filho et al. [50] showed that BAY 11-7082 decreased retinol induced redox-dependent NF-*κ*B-binding activity and p65 translocation from the cytoplasm to the nucleus. 

In our study, BAY 11-7082 administration caused an increase in tGSH and GSH concentrations when compared to the ET-1 group. These data indicate that pretreatment with BAY 11-7082 can protect the liver during oxidative stress in part by improving the concentration of reduced glutathione, which scavenges ROS and reduces their detrimental effects. Similarly, Kumar et al. [51] demonstrated that diabetic rats treated with BAY 11-7082 showed an increment in reduced glutathione (GSH) level and a decline in MDA content in sciatic nerve. GSH is a principal intracellular antioxidant-thiol compound and its reducing function is linked to maintain the redox homeostasis of cellular proteins [39]. Recently, Khan et al. [52] have indicated that inhibition of NF-*κ*B signaling pathway by caffeic acid resulted in an increased GSH content. Similarly, Chávez et al. [53] described that acetyl salicylic acid and ibuprofen inhibited NF-*κ*B activation and enhanced GSH, tGSH, and GSH/GSSG ratio in the liver fibrosis induced by carbon tetrachloride.

In our study, BAY 11-7082 has effectively suppressed TNF-*α* concentration in the liver tissue. We speculate that this effect resulted from the selective inhibition of the phosphorylation of IkB-*α* which decreased nuclear translocation of NF-*κ*B [54]. Our previous studies indicated that BAY 11-7082 reversed ET-1 induced oxidative lung injury [54]. Furthermore, Kumar et al. [51] reported that treatment with BAY 11-7082 diminished TNF-*α* level in diabetic rats. Also Sigala et al. [43] indicated that NF-*κ*B inhibition blunted the TNF-*α* in the diaphragm in rats. The decrease in TNF-*α* is at least in part caused by the inhibition of NF-*κ*B activity loop as NF-*κ*B plays the predominant role in TNF-*α* release. 

As BAY 11-7082 is an inhibitor of IkB*α* breakdown in our study it prevented ET-1-stimulated liver activation of p65 gene. Moreover, silencing of NF-*κ*B signaling resulted in less powerful downregulation of p21-cip gene than that in the ET-1 groups. Although inhibition of NF-*κ*B is beneficial regarding oxidative stress and proinflammatory compounds generation its protecting role on cell preservation is controversial. It was indicated that NF-B activation plays cytoprotective role in TNF-*α* induced hepatotoxicity [55]; however, the activation of NF-*κ*B may as well promote the survival of transformed hepatocytes, thus supporting malignancy and progression in cancer [56]. Therefore, this dual role of NF-*κ*B highlights the need of studying the molecular pathways that regulate the outcome of ET-1 stimulation to understand how NF-*κ*B exerts such distinct functions in the liver. 

On the contrary the stress-induced upregulation of p21 is incorporated with activation of p38 of MAPK signaling pathway and leads to apoptosis [57]. Hence, downregulation of p21 by BAY 11-7082 may have a protective effect on the liver cells.

In summary, our results have demonstrated that BAY 11-7082 is an effective compound that protects the liver from ET-1 induced oxidative stress damages. Concomitantly, BAY 11-7082 inhibited an excessive release of TNF-*α* caused by ET-1 which may result from reduced expression of NF-*κ*B and NF-*κ*B dependent p21-cip signaling pathways. Thus BAY 11-7082 might be considered as a clinical drug in the prevention and treatment of hepatic injury.

## Figures and Tables

**Table 1:** Effect of administration of BAY 11-7082 on oxidative stress parameters in liver homogenates of rats with endothelin-1-induced endotoxemia. Mean ± S.E.M. (*n* = 8).

Parameter	0.9% NaCl	ET-1 (1.25 mg/kg b.w.)	ET-1 (12.5 mg/kg b.w.)	BAY 11-7082 (10 mg/kg b.w.) + ET 1 (12.5 mg/kg b.w.)
TBARS (*μ*M)	5.96 ± 1.075	10.93 ± 1.16^###^	22.26 ± 2.43^∗#*∧∧*^	0.44 ± 0.22***
H_2_O_2_ (*μ*M)	1.44 ± 0.15	1.79 ± 0.06****	2.56 ± 0.15^∗*∧*##^	1.55 ± 0.19
GSH/GSSG ratio	7.13 ± 1.09	3.69 ± 0.27^∗#^	3.84 ± 1.02^####^	7.30 ± 0.46

TBARS: thiobarbituric acid-reactive substances; H_2_O_2_: hydrogen peroxide; redox status: GSH/GSSG ratio; *P < 0.001,**P < 0.01, ***P < 0.02, and ****P < 0.05 versus saline; ^#^ *P* < 0.001, ^##^ *P* < 0.01, ^###^ *P* < 0.02, and ^####^ *P* < 0.05 versus BAY 11-7082 + ET-1; ^*∧*^ *P* < 0.001, ^*∧∧*^ *P* < 0.01 versus ET-1 (1.25 mg/kg b.w.).

## Abbreviations

NF-*κ*B: Nuclear factor-kappaB
ROS: Reactive oxygen species
ET-1: Endothelin-1
TBARS: Thiobarbituric acid reactive substances
H_2_O_2_: Hydrogen peroxide
VEGF: Vascular endothelial growth factor
TNF: Tumor necrosis factor alpha
tGSH: Total glutathione
GSH: Reduced glutathione
GSSG: Oxidized glutathione
NADPH: Reduced form of nicotinamide adenine dinucleotide phosphate.
